# Population pharmacokinetic model to generate mechanistic insights in bile acid homeostasis and drug-induced cholestasis

**DOI:** 10.1007/s00204-022-03345-8

**Published:** 2022-07-25

**Authors:** Véronique M. P. de Bruijn, Ivonne M. C. M. Rietjens, Hans Bouwmeester

**Affiliations:** Division of Toxicology, Wageningen University and Research, Wageningen, 6708 WE The Netherlands

**Keywords:** Bile acids and salts, Cholestasis, Pharmacokinetics, PBK modeling

## Abstract

Bile acids (BA) fulfill a wide range of physiological functions, but are also involved in pathologies, such as cholestasis. Cholestasis is characterized by an intrahepatic accumulation of BAs and subsequent spillage to the systemic circulation. The aim of the present study was to develop physiologically based kinetic (PBK) models that would provide a tool to predict dose-dependent BA accumulation in humans upon treatment with a Bile Salt Export Pump (BSEP) inhibitor. We developed a PBK model describing the BA homeostasis using glycochenodeoxycholic acid as an exemplary BA. Population wide distributions of BSEP abundances were incorporated in the PBK model using Markov Chain Monte Carlo simulations, and alternatively the total amount of BAs was scaled empirically to describe interindividual differences in plasma BA levels. Next, the effects of the BSEP inhibitor bosentan on the BA levels were simulated. The PBK model developed adequately predicted the in vivo BA dynamics. Both the Markov Chain Monte Carlo simulations based on a distribution of BSEP abundances and empirical scaling of the total BA pool readily described the variations within and between data in human volunteers. Bosentan treatment disproportionally increased the maximum BA concentration in individuals with a large total BA pool or low BSEP abundance. Especially individuals having a large total BA pool size and a low BSEP abundance were predicted to be at risk for rapid saturation of BSEP and subsequent intrahepatic BA accumulation. This model provides a first estimate of personalized safe therapeutic external dose levels of compounds with BSEP-inhibitory properties.

**Supplementary Information:**

The online version contains supplementary material available at 10.1007/s00204-022-03345-8.

## Introduction

The main physiological role of bile acids (BAs) has long been recognized to be the emulsification of dietary lipids and lipid-soluble vitamins. Additionally, BAs are important signaling molecules between the gut microbes and the host and can regulate lipid, glucose and energy metabolism (Hylemon et al. 2009). Primary BAs are de novo synthesized in the liver via cytochrome P450 (CYP)-mediated oxidation of cholesterol. Subsequently, they are conjugated with taurine or glycine and either secreted into the common bile duct or into the liver sinusoids. The latter transport processes are mediated by several transporters in the liver, e.g., bile salt export pump (BSEP) and multidrug resistance protein 2 (MRP2) for BA secretion to the bile canaliculi and MRP3/4 for transport to the liver sinusoids. Via the bile, BAs are subsequently transported to the intestinal lumen, where secondary BAs are formed by microbial conversions. About 95% of the intestinal BAs are reabsorbed into the portal circulation and transported to the liver. This recycling process is called enterohepatic circulation (Farooqui and Elhence 2021; Jia et al. 2018). A disturbance in the BA homeostasis can distort the gut–liver axis and is associated with various pathologies, such as cholestasis. Cholestasis refers to a disrupted bile flow leading to the accumulation of BAs in the liver and subsequent spillage to the systemic circulation (Noor 2015). Cholestasis is among the most commonly observed adverse reactions in patients suffering from drug-induced liver injury (DILI) (Nunes et al. 2021). Currently available preclinical in vivo and in vitro screening methods are insufficiently able to predict DILI, or cholestasis more specifically (Olson et al. 2000; Vinken 2018; Xu et al. 2008).

Previously, inhibition of the hepatic BSEP has been identified as a risk factor involved in the development of cholestasis (Morgan et al. 2013). The importance of hepatic BSEP inhibition for cholestasis development is underlined by its recognition as a molecular initiation event (MIE) in the Cholestasis Adverse Outcome Pathway (AOP) (Vinken et al. 2013). Recently, an AOP network was established, visualizing the complexity of biological processes involved in the onset and development of human hepatotoxicity (Arnesdotter et al. 2021). The AOP network shows that BSEP inhibition is related to the key event (KE) bile acid accumulation, which in turn causes the release of pro-inflammatory mediators, activation of nuclear receptors/transcriptional change, and/or increased reactive oxygen species production. These KEs can lead directly or via multiple KEs to cholestasis. There are several compensatory mechanisms in the human liver to counteract hepatic BA accumulation involving the upregulation of other efflux transporters, e.g., OSTα/β and MRP3/4 (Gijbels et al. 2020; Jackson et al. 2016). Even though BSEP inhibition does not necessarily lead to cholestasis, it has been shown that integrating an export assay that measures the inhibition of BA export improves hepatotoxicity predictions compared to a cytotoxicity test in primary hepatocytes alone (Brecklinghaus et al. 2022).

Given the number of physiological processes involved in BA homeostasis, obtaining mechanistic insights in the synthesis, circulation and excretion of BAs is relevant to understand and predict BA-associated diseases, such as cholestasis. Computational physiologically based kinetic (PBK) modeling can be used to translate in vitro data to in vivo data (Louisse et al. 2017) and it provides a tool that can contribute to a mechanistic understanding of a xenobiotic’s distribution within the human body (Jones et al. 2015). In the current work we employ PBK modeling to elucidate interindividual differences that might determine susceptibility towards BSEP inhibition-mediated cholestasis. Bosentan, a drug used to treat pulmonary artery hypertension, has been shown to inhibit BSEP in a non-competitive nature in vitro and to cause DILI in 2–18% of the patients (Fattinger et al. 2001; Mano et al. 2007)*.* Given the potential of BSEP inhibitors to cause cholestasis and DILI, the aim of the present study was to develop a physiologically based kinetic (PBK) modeling facilitated approach linked with Markov Chain Monte Carlo simulations, that would provide a tool to predict dose-dependent BA accumulation in humans upon treatment with a BSEP inhibitor. A first proof-of-principle was developed using bosentan as the model BSEP inhibitor and glycochenodeoxycholic acid (GCDCA) as an exemplary BA.

## Methods

### Conceptual model

**Fig. 1:**
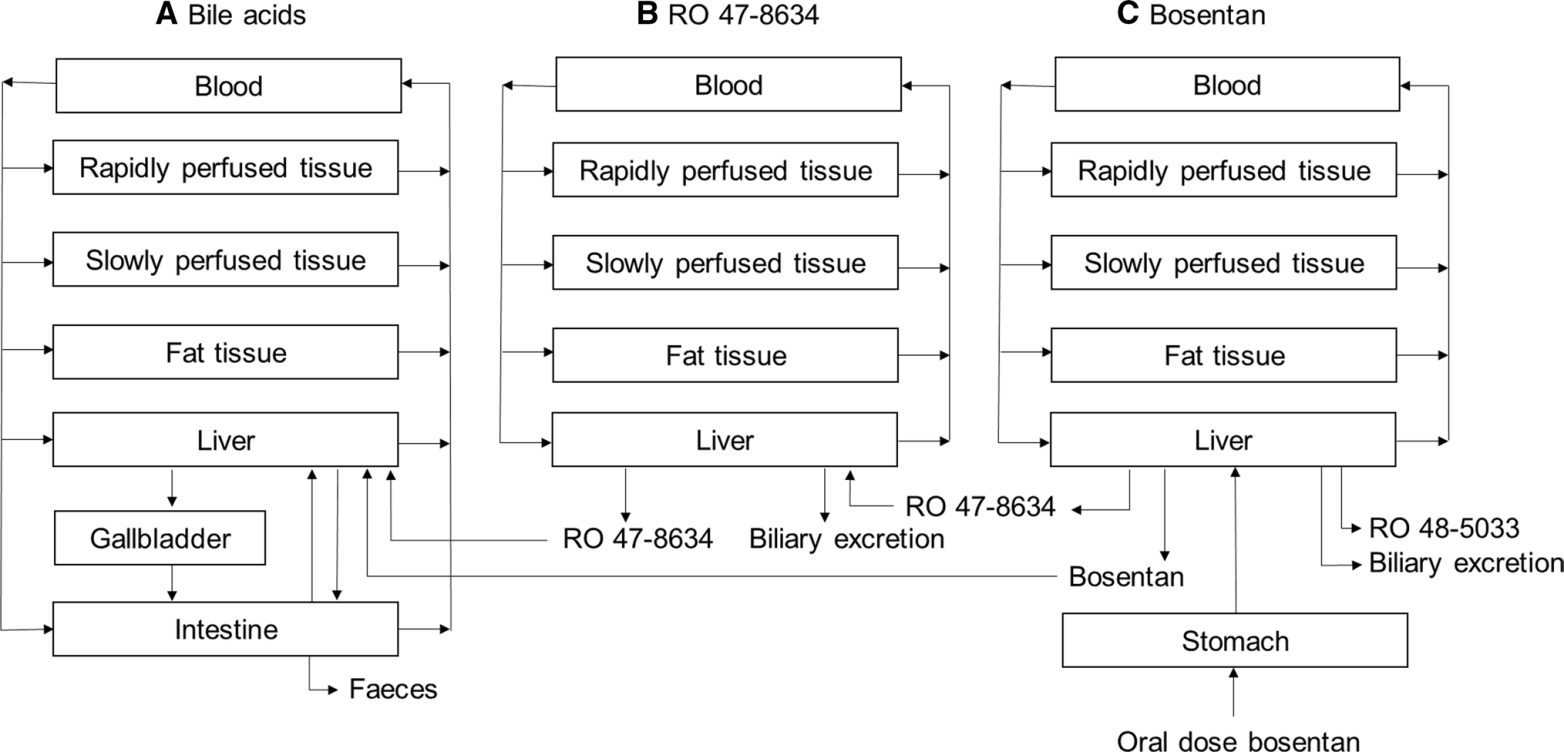
Schematic diagram of the PBK model for bile acids, bosentan and its active metabolite RO 47-8634. Submodels B and C were used to predict the intrahepatic free concentrations of bosentan and RO 47-8634 upon oral bosentan administration; these concentrations were subsequently used to predict the bosentan-induced effect on the bile acid concentrations using submodel A

Three PBK submodels were constructed: (A) a bile acid (BA) model, (B) a model to describe the kinetics of the active bosentan metabolite RO 47-8634 responsible for inhibition of BSEP and (C) a bosentan model (Fig. 1). Submodel A describes the synthesis, circulation and excretion of an exemplary BA, GCDCA, in a healthy reference individual. Submodels B and C were used to predict the free intrahepatic concentrations of bosentan and its active metabolite RO 47-8634. The intrahepatic concentrations were next used to predict the bosentan-induced inhibitory effects on BSEP-mediated efflux of BAs.

For modeling purposes, a lumped BA pool consisting of only GCDCA was assumed, supported by the fact that GCDCA is the most abundant BA in human serum (Bathena et al. 2013). Using a grouped BA pool enables us to keep the model complexity to a minimum, making the model easier to interpret and minimizing the risk of overfitting. The model consisted of separate compartments for liver, gall bladder, intestine, blood, rapidly perfused tissue, slowly perfused tissue and adipose tissue. The enterohepatic circulation was included by a circulation of GCDCA between the liver, gall bladder and intestine. GCDCA was de novo synthesized in the liver and excreted via the feces.

**Fig. 2:**
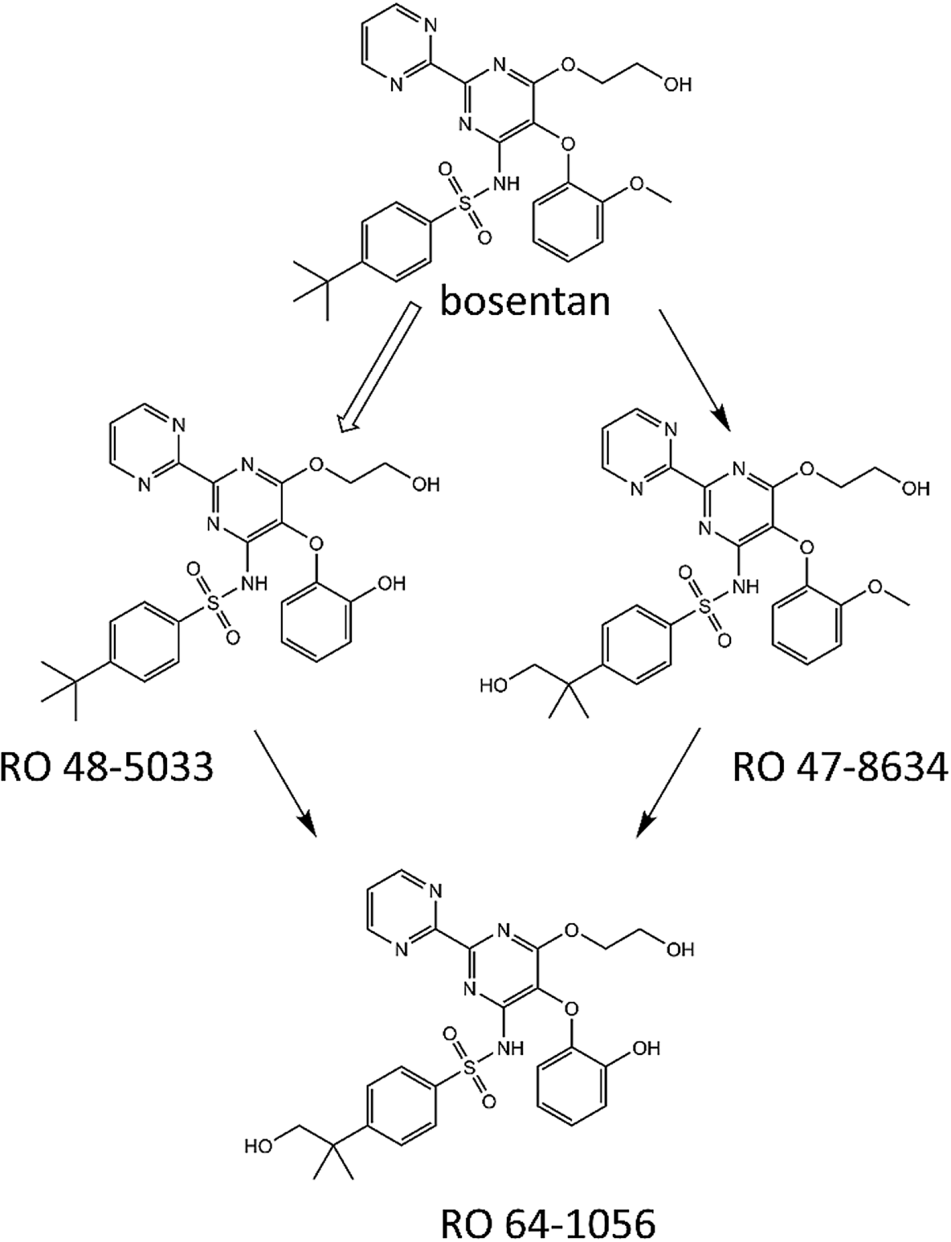
Metabolism of bosentan

The major metabolite of bosentan, RO 48-5033, is formed by hydroxylation at the t-butyl group of bosentan. Moreover, RO 47-8634 is generated by O-demethylation of the phenolic methyl ester of bosentan, and RO 64-1056 is generated by, respectively, O-demethylation and hydroxylation of the two mentioned bosentan metabolites, see Fig. 2. The major pathway of elimination of bosentan and its metabolites is hepatic metabolism followed by biliary excretion (Weber et al. 1999). Both bosentan and RO 47-8634 are able to inhibit BSEP-mediated transport of taurocholic acid in a non-competitive manner, while RO 48-5033 and RO 64-1056 had only limited effects on BSEP transport and are therefore not explicitly considered in a separate submodel (Fattinger et al. 2001). The PBK models for bosentan and RO 47-8634 consisted out of separate compartments for blood, rapidly perfused tissue, slowly perfused tissue, adipose tissue and liver.

### Physiologically based kinetic model of bile acid metabolism

Partition coefficients are important input parameters for PBK models and describe the relative distribution of a chemical between tissues and plasma at equilibrium within the organism. Tissue:plasma partition coefficients for GCDCA were calculated by a quantitative property–property relationship method described in literature (Rodgers and Rowland 2006) and obtained via the QIVIVE toolbox (Punt et al. 2020). The input parameters are summarized in Table 1. The total BA pool size in the reference individual was 3079 µmol. De novo synthesis in the liver was set to 46.8 µmol/h (Kullak-Ublick et al. 2004). To maintain the mass balance, fecal excretion of GCDCA was set equal to its de novo synthesis. GCDCA was actively transported from the liver to the common bile duct by BSEP following Michaelis–Menten kinetics. The BSEP-mediated efflux of GCDCA was described by the following equation (Eq. 1):

1 $$E = \frac{{V_{{{\text{max}},{\text{BSEP}}}} \,{*}\,\left[ {{\text{CVL}}} \right]}}{{K_{{m,{\text{BSEP}}}} + \left[ {{\text{CVL}}} \right]}}$$

where *E* is the BSEP-mediated efflux in µmol/h, *V*_max_ is the maximum efflux rate of GCDCA in blood in µmol/entire liver/hour, [CVL] the free concentration of BAs in µmol/L and *K*_*m*,BSEP_ the Michaelis–Menten constant in µmol/L for BSEP-mediated BA efflux.

**Table 1:** Physicochemical properties used to calculate the blood:tissue partition coefficients

GCDCA		Reference
p*K*_*a*_	3.77	Law et al. (2014)
log*P*	2.12	Roda et al. (1990)
MW	449.62	
Fraction unbound	0.01	Roda et al. (1982)
Blood:plasma ratio	0.55	Assumption (1-Hct), Cubitt et al. (2009)
*Bosentan*		
p*K*_*a*_	5.46	EMA (2004), Meyer (1996)
log*P*	3.1	EMA (2004), Meyer (1996)
MW	551.6	EMA (2004), Meyer (1996)
Fraction unbound	0.02	EMA (2004), Meyer (1996)
Blood:plasma ratio	0.6	EMA (2004), Meyer (1996)
RO 47-8634		
p*K*_*a*_	5.46	ALOGPS toolbox (Tetko et al. 2005)
log*P*	3.1	ALOGPS toolbox (Tetko et al. 2005)
MW	551.6	
Fraction unbound	0.02	Calculated, Lobell and Sivarajah (2003)
Blood:plasma ratio	0.55	Assumption (1-Hct), Cubitt et al. (2009)

The *V*_max_ and *K*_*m*_ for BSEP-mediated transport of GCDCA were obtained from a vesicular transport assay in a baculovirus-infected Sf9 system (Kis et al. 2009). The authors showed that the *V*_max_ values in the vesicular transport assay increased upon the addition of physiological levels of cholesterol, hence, these values were used in the current PBK model. The values were reported in µmol/min/mg BSEP. In order to scale the experimental value for *V*_max_ expressed in in µmol/min/mg BSEP to a value for the entire liver, an in vitro-in vivo extrapolation method was used based on absolute BSEP abundances. To this end, a scaling factor was calculated, as described in Eq. 2.

2 $${\text{SF}} = {\text{aBSEP}} \times {\text{MWBSEP }} \times {\text{Hep }} \times {\text{WL }} \times 60{ } \times 10^{ - 9} $$

where SF is the scaling factor in mg BSEP/entire liver, aBSEP is the BSEP abundance in pmol per 10^6^ hepatocytes, MWBSEP is the molecular weight of BSEP in g/mol, Hep is the hepatocellularity in 10^6^ cells/g liver, WL is the weight of liver in g, 60 is the number of minutes in an hour and 10^–9^ is used to convert pg to mg. As BSEP is a 140 kDa protein, a molecular weight of 140,000 g/mol was used.

It was assumed that half of the BAs in the common bile duct was secreted into the intestinal lumen, and the remaining half was stored in the gall bladder (Hofmann 1999). Gall bladder contractions were simulated three times per day, i.e., at 8:00, 12:00 and 16:00, assuming a meal consumption every four hours during daytime. Upon a gall bladder contraction, the entire gall bladder content was emptied in the intestinal lumen. GCDCA absorption from the intestine was assumed to follow first-order kinetics. The *k*_*a*_ value was obtained by fitting to in vivo data attained from Hepner and Demers (1977) and Ponz de Leon et al. (1978), which were scaled as described previously (Baier et al. 2019). Briefly, a percentage scaling factor was calculated from literature for scaling all datasets to the fraction of summed conjugated cholic, chenodeoxycholic and deoxycholic acid. Subsequently, the experimental data were multiplied by this scaling factor. Both datasets described plasma postprandial BA levels upon three subsequent meals in healthy subjects (in total 11 males and 3 females). Since the PBK model predicts whole blood concentrations and the in vivo data present plasma concentrations, the predicted whole blood concentrations were converted to plasma concentrations by dividing them by the blood:plasma ratio. The blood:plasma ratio of GCDCA was assumed to be 0.55 (1-hematocrit), which is a common assumption for acidic compounds when experimental data are lacking (Cubitt et al. 2009; Table 1). The fasting level of BAs in plasma was set to 2.4 µM (García-Cañaveras et al. 2012).

### Sensitivity analysis

To assess the influence of the parameters on the model outcome, a sensitivity analysis was performed for the *C*_max_ of BAs, bosentan and RO 47-8634. The BA submodel was assessed independently from the submodels for bosentan and metabolite RO 47-8634. Based on the method reported by Evans and Andersen (2000), the sensitivity coefficients (SCs) for the model parameters were calculated as follows:

3 $${\text{SC}} = \frac{{C^{\prime} - C}}{{P^{\prime} - P}}{ } \times P/C$$

where *C* indicates the initial value of the model output, *C*′ indicates the modified value of the model output resulting from an increase in the parameter value. *P* indicates the initial parameter value and *P*′ indicates the modified parameter value after a 5% increase of its value, keeping all other parameters at their original value.

### Two approaches towards describing interindividual differences in the BA pool

Two scenarios were applied to describe the variability in the systemic BA plasma levels in the reported in vivo data. In the first approach, the sensitivity analysis was used to identify parameters that have a strong influence on the *C*_max_ BA values and these parameters were multiplied with an empirical scaling factor “sens”. The identified parameters included parameters describing the total amount of BAs present, i.e., the amount of BA in a full gall bladder and the systemic fasting BA concentration in plasma. Therefore, we empirically scaled all the parameters contributing to the total BA pool size, i.e., dose in a full gall bladder, fasting concentration and de novo synthesis. As the fecal excretion was assumed to equal de novo synthesis to maintain the mass balance, this parameter was altered accordingly.

In the second approach, the variation that could occur in hepatic BSEP abundance in a Caucasian population was simulated using Markov Chain Monte Carlo simulations. Differences in BSEP abundance will result in an altered scaling factor (Eq. 2) and subsequently an altered *V*_max,BSEP_. For the Markov Chain Monte Carlo simulations, a total of 10,000 simulations were performed, where in each simulation BSEP abundance was randomly taken from a log-normal distribution derived from a meta-analysis of transporter abundances in liver tissue of healthy Caucasians (Burt et al. 2016). The distribution was truncated at ± 3 SD (Punt et al. 2016), excluding individuals with BSEP abundances three times higher or lower than the geometric mean. The mean, *µ*_*w*_ and standard deviation, *σ*_*w*_ describing the log-normal distribution of BSEP abundance were derived using Eqs. 4 and 5 (Ning et al. 2019; Zhang et al. 2007).

4 $$\mu_{w} = {\text{ln}}\frac{{\mu_{x} }}{{\sqrt {1 + {\text{CV}}_{x}^{2} } }}$$

5 $$\sigma_{w}^{2} = {\text{ln}}\left( {1 + {\text{CV}}_{x}^{2} } \right)$$

where *µ*_*x*_ is the mean BSEP abundance and CV_x_ is the coefficient of variation of BSEP abundance.

### Experimental data for postprandial bile acid levels

The maximum postprandial systemic BA values were compared with values reported in literature (see for relevant references Table 2). All studies included postprandial plasma BA levels in healthy adult subjects. The reported studies measured different BA conjugates; hence, data were normalized with a percentage scaling factor as described previously (Baier et al. 2019). If necessary, data were extracted from graphs using TechDig version 2.0. The study subjects received multiple meals, and each peak in postprandial BAs was assigned to a meal when possible.

**Table 2:** Details of experimental studies used for model validation

Gender	Age	Reference
5 females	24–53	Angelin and Björkhem (1977)
8 males, 6 females	22–56	Galeazzi et al. (1980)
3 males, 2 females	25–58	Gälman et al. (2005)
5 males, 3 females	24 ± 5 (mean ± SD)	Hepner and Demers (1977)
7 males, 4 males	27–61	Salemans et al. (1993)
6 males	24–39	Ponz de Leon et al. (1978)

### Physiologically based kinetic model of bosentan and RO 47-8634

PBK submodels B and C were constructed to derive the intrahepatic concentrations of bosentan and its active metabolite RO 47-8634. Eventually, these concentrations were used to simulate their inhibitory effect on BSEP-mediated efflux of BAs from the liver to the common bile duct. Both submodels B and C consisted of separate compartments for blood, rapidly perfused tissue, slowly perfused tissue, fat and liver, as shown in Fig. 2. A dosing regimen of 500 mg bosentan administered orally twice a day was selected, in line with available studies from which we extracted systemic in vivo plasma levels of bosentan and its active metabolite (Weber et al. 1999). Furthermore, systemic BA levels from volunteers following this dosing regimen were available (Fattinger et al. 2001). Bosentan administration was simulated at 8:00 and 20:00. The kinetic parameters for absorption of bosentan and biliary excretion of bosentan and RO 47-8634 were obtained by fitting to experimental plasma data obtained from Weber et al. (1999). The physicochemical properties of bosentan and RO 47-8634 were used to calculate partition coefficients using the QIVIVE toolbox (Punt et al. 2020). As experimental data were lacking for the p*K*_*a*_ and log*P* for RO 47-8634 these were predicted using the ALOGPS toolbox (Tetko et al. 2005). The fraction unbound of RO 47-8634 in plasma was calculated via the method described by Lobell and Sivarajah (2003) and obtained via the QIVIVE toolbox. The blood:plasma ratio of RO 47-8634 was assumed to be 0.55 (1-Hct) (Cubitt et al. 2009). The physicochemical properties of GCDCA, bosentan and RO 47-8634 are shown in Table 1.

The kinetic parameters for the metabolism of bosentan were obtained from a study with human liver microsome incubations (Sato et al. 2018). The metabolism consisted of a part following Michaelis–Menten kinetics and non-saturable metabolic clearance, as described in Eq. 6.

6 $$v = V_{{{\text{max}}}} \times { }\frac{{\left[ {{\text{CVL}}_{{{\text{bosentan}}}} } \right]}}{{Km + [{\text{CVL}}_{{{\text{bosentan}}}} ]}} + {\text{CL }} \times \left[ {{\text{CVL}}_{{{\text{bosentan}}}} } \right]$$

where *v* is the velocity of metabolism in µmol/h, *V*_max_ is the maximum velocity of metabolism in µmol/h, [CVL_bosentan_] is the free concentration of bosentan in liver in µmol/L, *K*_*m*_ is the Michaelis–Menten constant of metabolism in µmol/L and CL is the non-saturable metabolic clearance in L/h per 10^6^ cells. The reported kinetic parameters were expressed per mg microsomal protein and scaled to g liver, assuming 32 mg microsomal protein/g liver (Barter et al. 2007). The clearance was scaled from L/h per 10^6^ cells to L/h per g liver using a hepatocellularity of 99 $\times$ 10^6^ cells/g liver (Barter et al. 2007). To scale from g liver to entire liver, the kinetic parameters were multiplied by the liver weight. A liver weight of 20 $\times$ BW was used, i.e., 1400 g of liver for a 70 kg individual (Soars et al. 2002).

### Inhibition

Bosentan and RO 47-8634 inhibit BSEP-mediated BA efflux in a non-competitive nature (Fattinger et al. 2001). Hence, the Michaelis–Menten equation in the model that represents BSEP-mediated BA efflux from the liver was modified to include the non-competitive type of inhibition by the introduction of a modulation factor (1 + [CVL_bosentan_]/*K*_*i*,bosentan_ + [CVL_RO 47-8634_]/*K*_*i*,RO 47-8634_). The resulting equation for BSEP-mediated BA efflux is then as follows:

7 $$E = V_{{{\text{max}},{\text{BSEP}}}} /\left( {1 + \left[ {{\text{CVL}}_{{{\text{bosentan}}}} } \right]/K_{{i,{\text{bosentan}}}} + \left[ {{\text{CVL}}_{{{\text{RO }}47{ - }8634}} } \right]/K_{{i,{\text{RO }}47{ - }8634}} } \right){*}\left[ {{\text{CVL}}} \right]/\left( {K_{{\text{m,BSEP}}} { } + { }\left[ {{\text{CVL}}} \right]} \right)$$

where *E* is the BSEP-mediated efflux of BAs from the liver (µmol), *V*_max,BSEP_ is the maximum rate of BSEP-mediated BA efflux from the liver in µmol/h, [CVL_bosentan_] is the free concentration of bosentan in µmol/L in the liver, *K*_*i*,bosentan_ is the inhibition constant for inhibition of the BSEP-mediated BA efflux by bosentan in µmol/L, [CVL_Ro 47-8634_] the free concentration of RO 47-8634 in µmol/L in the liver, *K*_*i*,RO 47–8634_ is the inhibition constant for inhibition of the BSEP-mediated BA efflux by RO 47-8634 in µmol/L, [CVL] the free concentration of BAs in µmol/L in the liver and *K*_*m*,BSEP_ is the Michaelis–Menten constant of BSEP-mediated BA efflux in µmol/L in the liver. Bosentan and RO 47-8634 inhibited BSEP-mediated transport of taurocholic acid with *K*_*i*_ values of 12 and 8.5 µmol/L, respectively (Fattinger et al. 2001). In the absence of data, it was assumed that the *K*_*i*_ values for the competition with GCDCA equaled those for taurocholic acid.

The differential model equations were encoded and solved using Berkeley Madonna 10.2.8 (UC Berkeley, CA, USA) using the auto-step size algorithm. The full model code is presented in Supplementary file II. Pre- and post-treatment BA levels were compared with a Wilcoxon signed-rank test using R version 4.0.2 (R Core Team 2020). A *p* value < 0.05 was considered statistically significant.

## Results

**Fig. 3:**
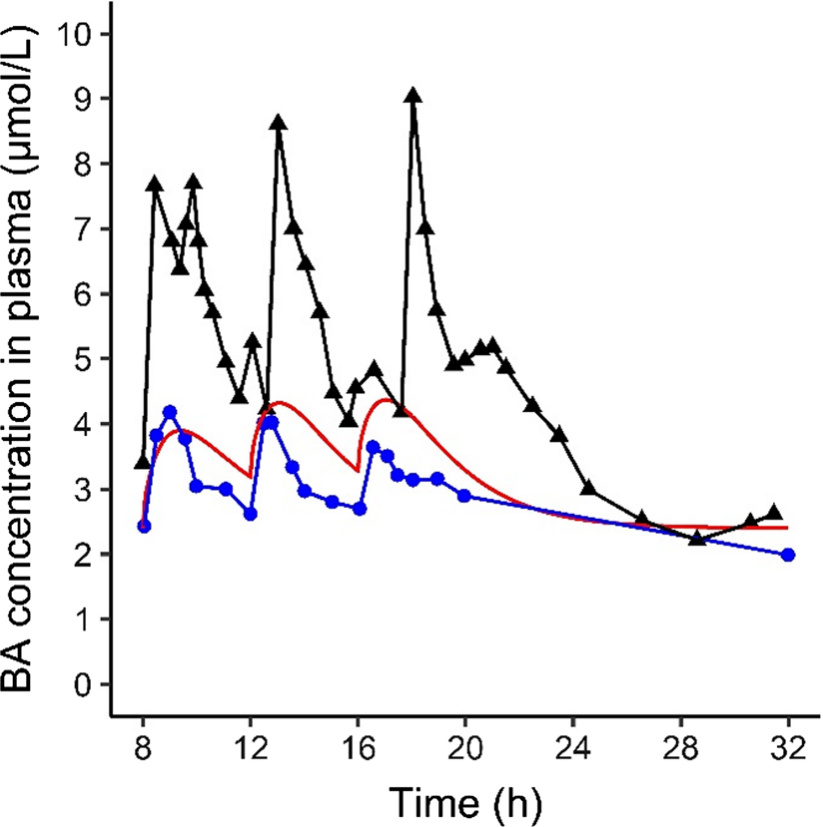
Predicted and observed plasma BA levels in human subjects. Subjects fasted overnight and meals were simulated at 8:00, 12:00 and 16:00. In vivo data were retrieved from Hepner and Demers (1977) (blue circles) and Ponz de Leon et al. (1978) (black triangles). The simulation is visualized in red

**Fig. 4:**
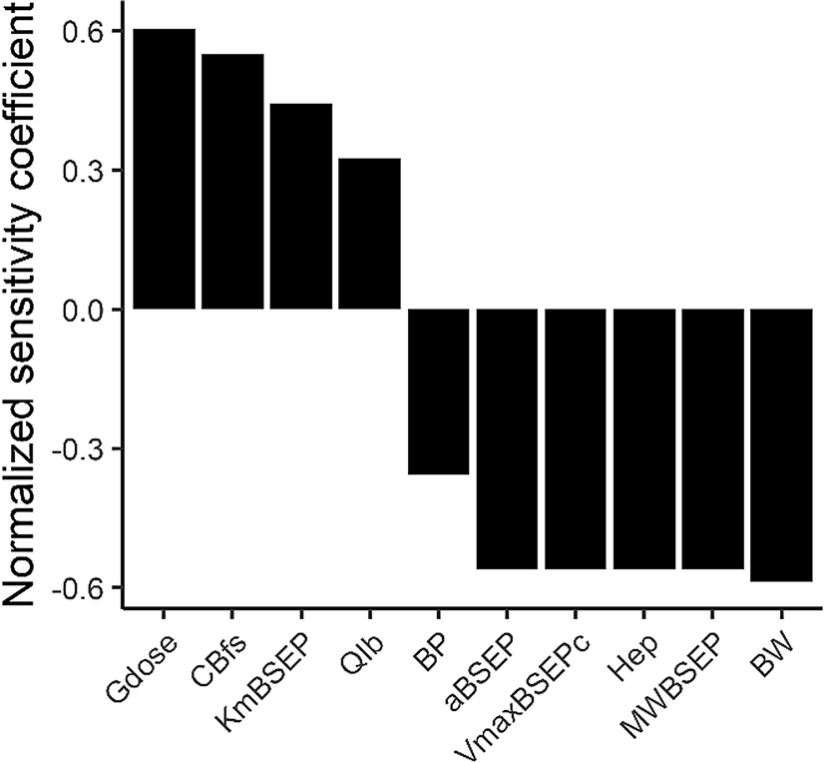
Sensitivity analysis of the influence of the PBK model parameters on the predicted maximal systemic BA concentration in plasma. Only parameters with an absolute normalized sensitivity coefficient > 0.1 are shown. Gdose = dose in a full gallbladder, CBfs = systemic plasma concentration in fasting state, KmBSEP = Michaelis–Menten constant for BSEP-mediated BA efflux from the liver, QIb = fraction of bile flow transported directly from liver to intestinal lumen via common bile duct reference, BP = blood:plasma ratio, aBSEP = BSEP protein abundance, VmaxBSEPc = maximal BSEP-mediated BA efflux rate, Hep = hepatocellularity, MWBSEP = molecular weight of BSEP, BW = body weight

A computational model describing the synthesis, circulation and excretion of bile acids (BAs) was developed for a healthy reference individual (Fig. 3). In the simulations subjects fasted overnight and meals were simulated at 8:00, 12:00 and 16:00. Upon meal ingestion, the gall bladder was contracted, resulting in a peak in the systemic BA levels. The prediction was compared with two experimental data sets available in the literature showing postprandial BA levels (Hepner and Demers 1977; Ponz de Leon et al. 1978) (Fig. 3). The predicted systemic maximum BA concentration in plasma (*C*_max_) value was 4.4 µM, which is in line with the in vivo data reported by Hepner and Demers (1977). The *C*_max_ reported by Ponz de Leon et al. (1978) is more than twofold higher (Fig. 3). The established PBK model was utilized to explore mechanistic explanations for these differences in the in vivo data. For this, a sensitivity analysis was performed to identify parameters that have a strong influence on the model outcome, i.e., the systemic plasma BA concentration. The sensitivity analysis (Fig. 4) revealed that parameters determining the amount of BA present in the body at the start of the simulations, i.e., the amount of BAs in a full gall bladder (Gdose) and systemic fasting concentration (CBfs) showed a strong positive influence on the model outcome (*C*_max_). Parameters determining the scaled maximum rate of BSEP-mediated BA efflux had a strong negative influence on the *C*_max._

### Markov Chain Monte Carlo simulations for BSEP abundances

To describe the interindividual variability in the systemic BA levels observed in vivo, we employed two scenarios. In the first approach, the BSEP abundances were randomly drawn from a log-normal distribution to simulate its variability in a virtual population using Markov Chain Monte Carlo simulations. In the second scenario, we scaled the total BA pool size with an empirical scaling factor informed by the results from the sensitivity analysis (Fig. 4).

**Fig. 5:**
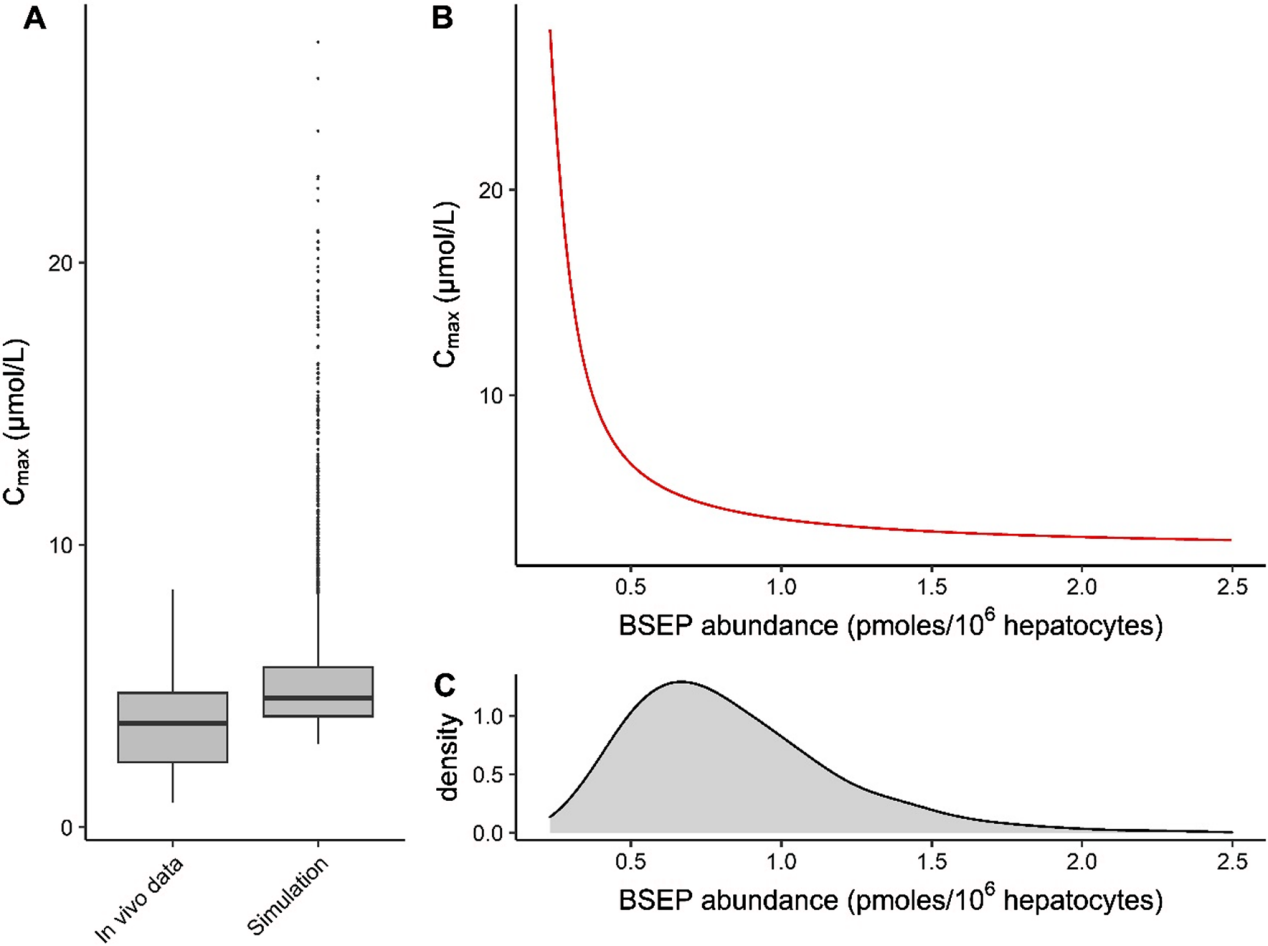
**a** Predicted and observed BA concentrations in plasma. BSEP abundances were drawn randomly from a log-normal distribution using Markov Chain Monte Carlo simulations. The details for the distribution were obtained from Burt et al. (2016). 10,000 iterations were performed, 18 iterations were excluded because BSEP abundances exceeded ± 3 SD. For details about the in vivo data set, see “Methods”. **b** Relationship between simulated C_max_ and BSEP abundance. **c** Density plot of BSEP abundances used for the Markov Chain Monte Carlo simulations

To study the effect of interindividual differences in absolute BSEP abundances on the BA homeostasis, a set of 10,000 Markov Chain Monte Carlo simulations were performed. In these simulations, BSEP abundances were randomly sampled from a log-normal distribution and used for in-vitro-to-in-vivo scaling of the experimentally obtained *V*_max_ for BSEP-mediated efflux of BAs. The predicted *C*_max_ values were compared with observed *C*_max_ values (Fig. 5a). The predicted and observed data for healthy individuals gave comparable median values. Figure 5b displays that individuals with a low BSEP abundance have very high *C*_max_ values, while the probability of an individual having such a low BSEP abundance is simulated to be small (Fig. 5c).

### Empirical scaling of the total bile acid pool size

**Fig. 6:**
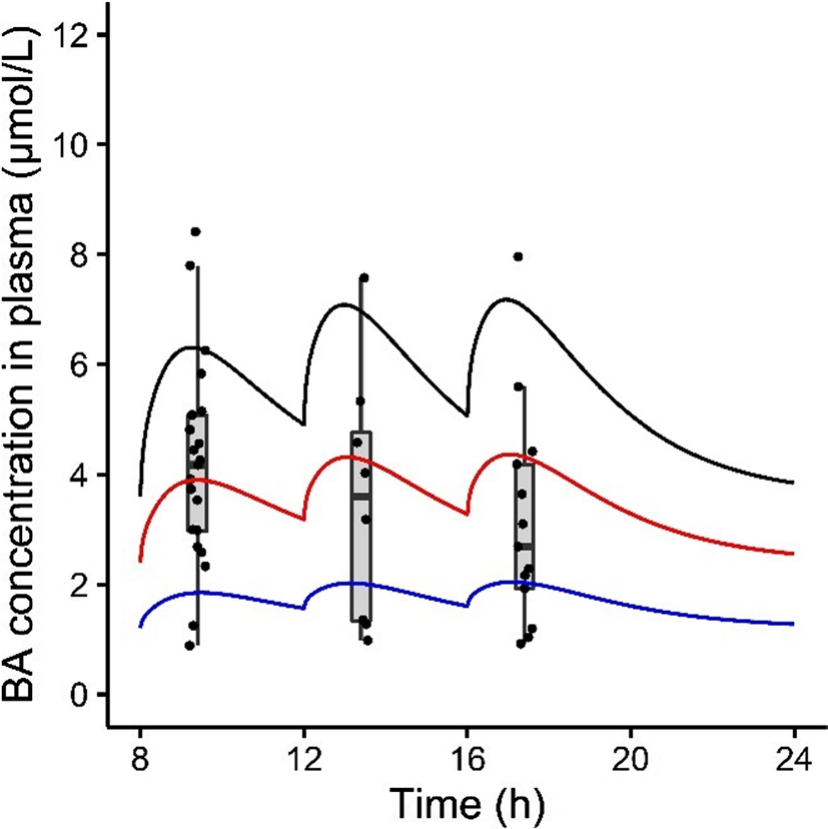
The effect of the empirical scaling factor sens on predicted plasma BA levels in human subjects. Subjects fasted overnight and meals were simulated at 8:00, 12:00 and 16:00. The dose in a full gall bladder, the systemic fasting concentration, the de novo synthesis and the fecal excretion were multiplied with a certain sens value (0.5, 1 or 1.5) to model the effects of different total BA pool size. The total pool size in the reference individual (sens value = 1) was 3079 µmol. The boxplots and points represent observed peak plasma BA concentrations after three meals reported in literature (see “Methods”). Blue = sens value 0.5, red = sens value 1, black = sens value 1.5

The sensitivity analysis revealed that the amount of BAs in a full gall bladder (Gdose) and the systemic fasting concentration (CBfs) had a large influence on the systemic BA peak concentrations. Therefore, we altered the total BA pool size with an empirical scaling factor “sens” (sensitivity). The total BA pool size is governed by the amount of BAs in a full gall bladder, the systemic fasting concentration and the de novo synthesis. These parameters were multiplied with a certain value sens (0.5, 1 or 1.5). As the fecal excretion was assumed to equal de novo synthesis in order to maintain the mass balance, this parameter was altered accordingly. The total BA pool size in the reference individual was 3079 µmol. Using the respective empirical scaling factors sens this value amounted to 1540, 3079 and 4619 µmol. In Fig. 6, the effect of the empirical scaling factor sens on the systemic plasma BA concentrations is visualized and compared with available in vivo data for the *C*_max_ after each meal. Each of the factors for sens resulted in predictions within the boundaries of the reported in vivo data. A sens value of 1 gives a prediction within the interquartile range (IQR) of the in vivo data; a sens values of 1.5 provides a prediction above the IQR; a sens value of 0.5 predicted systemic BA plasma concentrations below the IQR after meal 1 and at the lower end of the IQR after meal 2 and 3. Furthermore, the increases in the amount of BAs in each of the organs upon introduction of the empirical scaling factors were evaluated (Table 3). With a sens value of 1.5, the intrahepatic BAs showed the largest increase relative to the reference individual (sens value 1) compared to the other organs.

**Table 3:** Fold change in the amount of BAs in compartments of the PBK model upon empirical scaling of the total BA pool relative to a reference individual (sens value = 1). The total amount of BAs was multiplied with a factor 0.5 or 1.5 (sens value = 0.5 or 1.5, respectively)

	Fold change	
	Sens value 0.5	Sens value 1.5
Adipose tissue	0.4	1.8
Blood	0.4	1.8
Gall bladder	0.5	1.5
Intestinal lumen	0.5	1.5
Intestinal tissue	0.4	1.8
Liver	0.4	2.1
Rapidly perfused tissue	0.4	1.8
Slowly perfused tissue	0.4	1.8

### Submodels bosentan and metabolite RO 47-8634

**Fig. 7:**
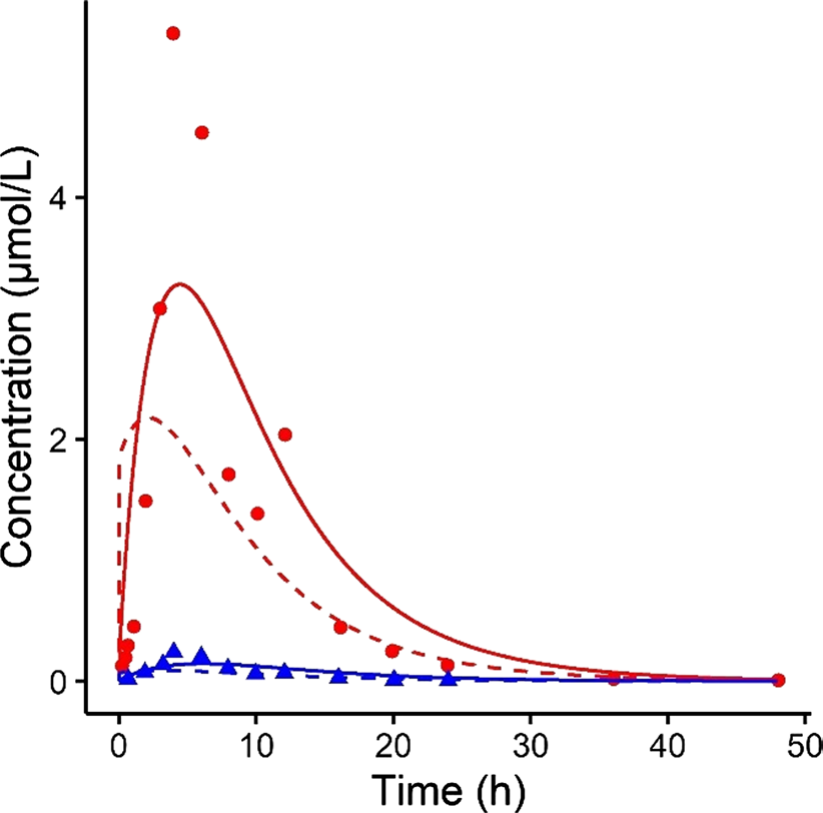
Predicted and observed concentrations of bosentan and RO 47-8634 upon oral administration of 500 mg bosentan. Circles and triangles represent experimental data in plasma obtained from Weber et al. (1999), the solid lines represent the predictions in plasma and the dashed lines the prediction of free concentration in the liver. Red = bosentan, blue = RO 47-8634

After the establishment of the PBK model for BAs, the submodels describing the distribution of the cholestatic drug bosentan and its metabolite RO 47-8634 upon oral administration of 500 mg were developed. The results of the simulation for the systemic levels (solid lines) and the experimental data (dots and triangles) (Weber et al. 1999) are displayed in Fig. 7. The experimental plasma data were 1.7-fold higher than the simulations for both bosentan and RO 47-8634. These PBK submodels were used to predict the free intrahepatic concentrations of bosentan and RO 47-8634 (dashed lines), and subsequently used to predict the inhibitory effect on BSEP-mediated BA efflux from the liver. The sensitivity analysis revealed that the fraction absorbed (Fa) and oral dose of bosentan (ODOSEmg) had the strongest positive influence on the *C*_max_. The strongest negative influence on the model outcome was observed for the fraction of liver tissue (VLc). The normalized sensitivity coefficients are displayed in Supplementary Figure S1.

**Fig. 8:**
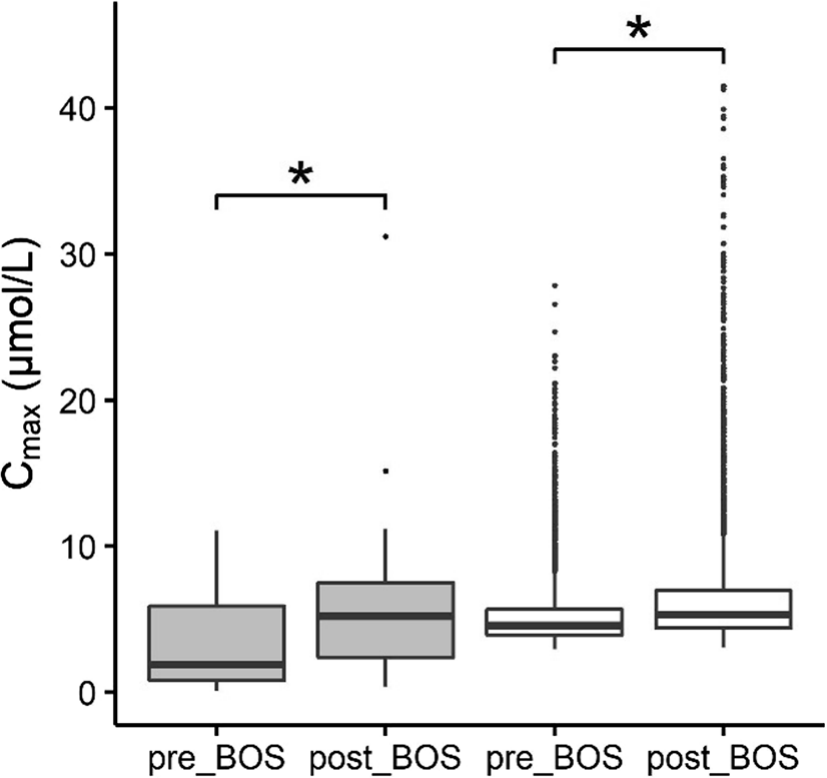
Predicted and observed BA concentrations in the plasma of controls and individuals administered with bosentan 500 mg twice a day. BSEP abundances were drawn randomly from a log-normal distribution using Markov Chain Monte Carlo simulations. 18 and 15 simulations were discarded because BSEP abundances exceeded ± 3 SD for controls and bosentan-treated individuals, respectively. Gray fill: in vivo data and statistical results retrieved from Fattinger et al. (2001); white fill: simulated data

**Fig. 9:**
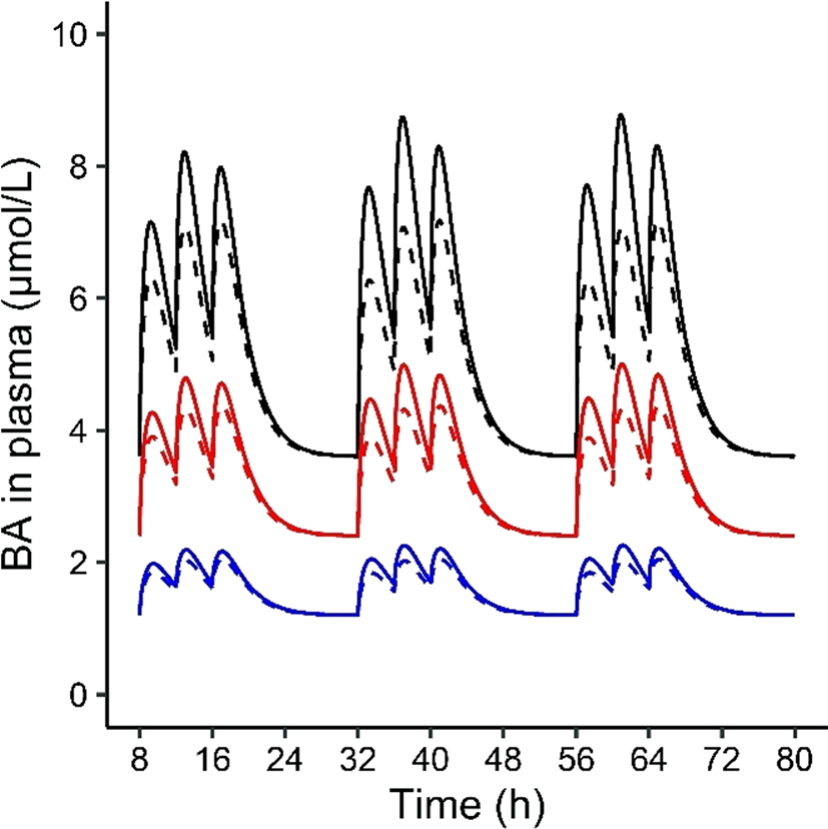
The effects of oral bosentan administration (500 mg twice per day) on systemic BA plasma values. Subjects fasted overnight and meals were simulated at 8:00, 12:00 and 16:00. The dose in a full gall bladder, the systemic fasting concentration, the de novo synthesis and the fecal excretion were multiplied with a certain sens value (0.5, 1 or 1.5) to model the effects of different total BA pool size. The total pool size in the reference individual (sens value = 1) was 3079 µmol. Dashed line = no bosentan; solid line = bosentan administration. Blue = sens value 0.5, red = sens value 1, black = sens value 1.5

The effect of repeated bosentan administration on systemic BA levels was simulated for a reference individual, as well as for a virtual population using the Markov Chain Monte Carlo simulations for BSEP abundances (Fig. 8) and with the empirical scaling factor sens (Fig. 9). Fattinger et al. (2001) reported the highest DILI incidence upon a dosing regimen of 500 mg bosentan twice per day, thus the same dosing regimen was used in the simulations. Bosentan was simulated to be administered orally at 8:00 and 20:00, the first dose being administered along with breakfast. The model predicted that the systemic *C*_max_ values of bosentan and RO 47-8634 reached stable levels after three days of repeated bosentan dosing, respectively. Therefore, postprandial kinetics of BA levels during three days of oral bosentan administration appeared sufficient to obtain insight in the effect of repeated bosentan dosing on BA kinetics. Figure 8 shows the simulated and observed effect of bosentan treatment on the BA plasma concentrations in a virtual population with different BSEP transporter abundances, and a population of healthy individuals who developed liver injury during the clinical trial using bosentan (18% of the participants) (Fattinger et al. 2001), respectively. The pre- and post-treatment BA levels were reported in the clinical trial; likewise, we report healthy controls and post-treatment levels. Both the observed and simulated data displayed a significant increase in systemic BA concentrations upon repeated bosentan administration.

Bosentan treatment increased the maximum postprandial BA level for the reference individual, as well as for the simulations with sens values of 0.5 and 1.5 (Fig. 9). The largest increase was observed with a sens value of 1.5. Maximum concentrations of BA in the liver are reported in Table 4; the largest increase was observed with a sens value of 1.5.

**Table 4:** Maximal simulated hepatic BA concentrations in healthy controls and bosentan-administered individuals (500 mg twice per day)

Sens value	Maximal hepatic BA concentration (nmoles/g tissue)		
	0.5	1	1.5
Control	0.2	0.5	1.1
Bosentan	0.3	0.8	1.8

## Discussion

The current study presents a PBK model describing the synthesis, circulation and excretion of bile acids (BAs), using GCDCA as the model BA, in healthy and bosentan-treated individuals. Bosentan is known to inhibit the efflux of BAs from the liver to the common bile duct by non-competitive inhibition of the hepatic BSEP transporter, which can lead to intrahepatic accumulation of BAs and their subsequent spillage to the systemic circulation (Fattinger et al. 2001; Mano et al. 2007). The current study showed that especially a large total BA pool size and a low BSEP abundance increase an individual’s susceptibility towards BSEP inhibition-mediated toxicity.

Postprandial plasma BA levels were simulated and compared with in vivo data reported in literature. The reported in vivo data show a ninefold variation between individuals (Angelin and Björkhem 1977; Galeazzi et al. 1980; Gälman et al. 2005; Hepner and Demers 1977; Ponz de Leon et al. 1978; Salemans et al. 1993). Our simulations predicted BA levels that were well within the reported range and followed a similar peak pattern as reported in literature (Hepner and Demers 1977; Ponz de Leon et al. 1978). Next, we employed two scenarios to identify plausible causes of the interindividual differences, and identify risk factors for the development of cholestasis. In the first approach, we incorporated physiologically relevant BSEP abundances in our PBK model for quantitative in-vitro-to-in-vivo extrapolations of *V*_max,BSEP_. The abundances were randomly drawn from a log-normal distribution (Burt et al. 2016) using Markov Chain Monte Carlo simulations. Our results revealed that the BA *C*_max_ values in the plasma of individuals with low BSEP abundances reach very high levels (Fig. 5b); 0.9% of our simulated population has abnormal systemic BA values of over 15 µM. As we identified low BSEP abundances as a risk factor for the development of cholestasis, it is important for future safety assessments of BSEP-inhibitory pharmaceuticals to get an accurate estimate of the prevalence of low abundances. Not only low transporter abundance, but also low transporter functionality as a result of inborn mutations should be considered as a cause for increased hepatic BA concentrations and susceptibility towards BSEP inhibition-mediated toxicity. Carriers of progressive familial intrahepatic cholestasis 2 (PFIC2) or benign recurrent intrahepatic cholestasis 2 (BRIC2) have a higher risk of developing cholestasis during their lifetime. Both PFIC2 and BRIC2 are caused by polymorphisms of the BSEP-coding gene which leads to a dysfunctional BSEP protein. PFIC2 and BRIC2 are estimated to occur in about 1 per 50,000 to 1 per 100,000 childbirths (Geethalakshmi and Mageshkumar 2014). In BRIC2 carriers, usually, a basal functionality of BSEP remains, while PFIC2 is more severe and patients ultimately need liver transplantation as they develop cholestasis, progressive liver fibrosis, cirrhosis and end-stage liver disease (Srivastava 2014). With accurate kinetic data for the BA transport remaining in BRIC2 or PFIC2 carriers, the current model could be used to set safe external dose levels of xenobiotics for these individuals.

The sensitivity analysis revealed that the BA levels in a full gall bladder and the fasting concentration of BAs contributed strongly to the predicted systemic BA levels, and thus could be a major determinant of the interindividual variability. Based on these results, we introduced an empirical scaling factor that modified the total amount of predicted BAs present in the body. We found that relatively small factors of 0.5 and 1.5 could predict the lower and upper range of the reported in vivo data. Our reference individual has a total BA pool of 3079 µmol; which is slightly below reported values (3672–9374 µmol (Beuers et al. 1992; Koopman et al. 1988), see Supplementary file I Table S1 for the conversion to model units). This discrepancy can be explained because the BA concentration in the liver, intestine, fat, slowly and rapidly perfused tissue were set to 0 at the beginning of the simulations, while in vivo BAs circulate through these tissues. This could be overcome by carefully defining the initial state in the relevant organs, although this is challenging as experimental data are sparse for some compartments of the human body that are inaccessible. The empirical scaling factor 1.5 resulted in a total pool size well within the physiological range, while the maximal hepatic BA levels were below the physiological range (16–67 nmoles/g tissue (Aranha et al. 2008; García-Cañaveras et al. 2012)). This indicates that in our predictions a (too) large fraction was secreted from the liver or a (too) small fraction of the BAs was absorbed from the intestinal lumen, causing low predictions hepatic BA levels. Refining the PBK model with more mechanistic insights in intestinal and hepatic BA uptake might improve the predictions for intrahepatic BA levels. Interestingly, a 1.5-fold increase in the amount of BAs present in the body led to a 2.1-fold and 1.8-fold increase in the hepatic and systemic blood BA concentrations, respectively (Table 3). Along with the relatively small increase in BA levels observed in the intestinal lumen, this indicates saturation of the BSEP-mediated efflux of BAs from the liver to the common bile duct. Via diffusion the BAs deposit from the liver to the remaining organs and the systemic circulation, resulting in elevated systemic concentrations. Hence, a large BA pool can lead to saturation of BSEP and is thereby a risk factor for the development of drug-induced cholestasis.

The next step in our study was to establish a PBK model for bosentan and its metabolite RO 47-8634 to estimate their intrahepatic concentrations, and subsequently their inhibitory effect on BSEP-mediated BA transport. Our PBK model gave a good prediction of the bosentan and RO 47–8634 concentrations in plasma (Fig. 7), given that the simulated C_max_ was within the twofold cut-off value that is commonly requested within a regulatory context (Peters and Dolgos 2019). Therefore, the bosentan model was considered fit for purpose to predict to intrahepatic bosentan and RO 47-8634 levels and use these as input in the BA PBK model. Upon the introduction of bosentan’s inhibitory effect on BSEP-mediated transport in the BA model, the systemic BA levels increased, as was also reported in vivo (Fattinger et al. 2001). It should be noted that the in vivo study only measured pre- and post-treatment BA levels in individuals who developed DILI, whereas the simulated population was a healthy population. However, as the experimental and simulated pre-treatment BA levels were comparable, we assume that the individuals who developed liver injury throughout the bosentan treatment did not have a specific predisposition towards developing DILI. In line with the in vivo data, very high BA levels of up to 42 µM were observed in the simulations upon bosentan treatment. Interestingly, the simulations with the empirical scaling factor of 1.5 showed a larger increase in systemic BA levels than what was predicted for the reference individual upon bosentan treatment (Fig. 9). This larger increase further supports that BSEP-mediated BA efflux is more rapidly saturated in the individuals with a 1.5-fold scaled and thus higher total BA pool. Furthermore, plasma C_max_ values were strongly increased in individuals with low BSEP abundances. Hence, either a large total BA pool size or a low BSEP abundance can result in an overloaded BSEP transport, which in turn results in excessively high hepatic and potentially toxic BA levels, especially when a BSEP-inhibiting drug is administered.

The current PBK model condensed a complex biological system to a relatively simple set of equations, with proved to adequately predict physiological responses. The BA pool was considered as a lumped pool, with GCDCA as an exemplary BA, although in vivo the BA pool consists of various BAs, with each different kinetics and physicochemical properties. Computational models describing different BA species in the human body have been established recently (Sips et al. 2018; Voronova et al. 2020). However in contrast to these studies, in the current PBK model all but one parameters were derived experimentally. We consider this as a major strength of the current model which could only be accomplished by reducing the model complexity. We limited the number of BAs included in the model, as experimental kinetic data are simply not available for all BAs. The current model can be easily extended towards individual BAs as soon as relevant kinetic data become available. Furthermore, in the current approach basolateral transport was assumed to be completely diffusion-mediated and active transport of BAs by MRP3, MRP4 or OSTα/β was not yet considered. The expression of these transporters is low under normal conditions (Beaudoin et al. 2020; Vinken et al. 2013), and hence, incorporation of these transporters is expected to not have a substantial effect on the intrahepatic or systemic BA levels of healthy individuals. Nevertheless, under cholestatic conditions, MRP3/4 and OSTα/β are upregulated (Gijbels et al. 2020; Vinken et al. 2013), by that counteracting the intrahepatic accumulation of BAs. Experimental data regarding basolateral transporter affinity and regulation of its expression are lacking to date, but would improve the accuracy of the simulations of cholestatic individuals.

In conclusion, the current PBK model provided novel mechanistic insight into BA homeostasis and the consequences of BSEP inhibition and helps to, e.g., identify rate-limiting processes or risk factors towards developing BA related liver disease. With this, the PBK modeling approach serves as a vigorous instrument to understand the BA homeostasis without the need for animal testing. We identified that individuals with low functional BSEP abundances or a large BA pool are susceptible to BSEP-mediated cholestasis. In these individuals, BSEP-mediated hepatic BA efflux is rapidly saturated upon BSEP inhibition, causing elevated and potentially hepatotoxic BA concentrations. Since the current PBK model is data-driven (i.e., most input parameters are derived experimentally), it is suitable to extrapolate to other situations or individuals. A powerful application of the coupled bosentan–BA model is the potential to predict dose-relationships for specific individuals, e.g., PFIC2 or BRIC2 carriers, or to estimate safe therapeutic doses for an entire population, including the most sensitive individuals. The approach developed can easily be extended to other pharmaceuticals, for which the needed model input parameters are typically known.

## Supplementary Information

Below is the link to the electronic supplementary material.

Supplementary file1 (DOCX 86 kb)

## Abbreviations

AOP: Adverse outcome pathway
BA: Bile acid
BSEP: Bile salt export pump
*C*_max_: Maximum concentration in plasma
DILI: Drug-induced liver injury
GCDCA: Glycochenodeoxycholic acid
Hct: Hematocrit
IQR: Interquartile range
KE: Key event
PBK: Physiologically based kinetic
MRP2/3/4: Multidrug resistance protein 2/3/4
MIE: Molecular initiating event
OSTα/β: Organic solute transporter alpha/beta
