# Supplementary material for: Population pharmacokinetic model to generate mechanistic insights in bile acid homeostasis and drug-induced cholestasis
Source: Arch Toxicol. 2022 Jul 25;96(10):2717–30. doi: 10.1007/s00204-022-03345-8 (PMC9352636; doi:10.1007/s00204-022-03345-8)
Supplement: Supplementary file 1 — Supplementary file1 (DOCX 86 kb) [file 204_2022_3345_MOESM1_ESM.docx]

# Supplementary file I

Table S1 Total BA pool sizes; scaled to presence of conjugates of CA, CDCA and DCA and to a 70 kg individual.

| Koopman, 1988 |  |  |  |  |  |
| --- | --- | --- | --- | --- | --- |
| CA (µmol/kg bw) | CDCA (µmol/kg bw) | total (µmol/kg bw) | total (µmol/70 kg) | scaled (µmol/70 kg) | |
| 11.1 | 19.8 | 30.9 | 2163 | 4023 |  |
| 18.4 | 9.8 | 28.2 | 1974 | 3672 |  |
| 36.5 | 35.5 | 72 | 5040 | 9374 |  |
| 42.5 | 25.1 | 67.6 | 4732 | 8802 |  |
| 19.2 | 20.5 | 39.7 | 2779 | 5169 |  |
| 14.3 | 24.9 | 39.2 | 2744 | 5104 |  |
| 26.8 | 25.7 | 52.5 | 3675 | 6836 |  |
|  |  |  |  |  |  |
| Beuers, 1992 |  |  |  |  |  |
| DCA (µmol/kg bw) | CDCA (µmol/kg bw) | total (µmol/kg bw) | total (µmol/70 kg) | scaled (µmol/70 kg) | |
| 16.3 | 22.4 | 38.7 | 2709 | 3684 |  |
| 42.1 | 18.9 | 61 | 4270 | 5807 |  |
| 30.2 | 13.6 | 43.8 | 3066 | 4170 |  |
| 42.2 | 24.9 | 67.1 | 4697 | 6388 |  |
|  |  |  |  |  |  |


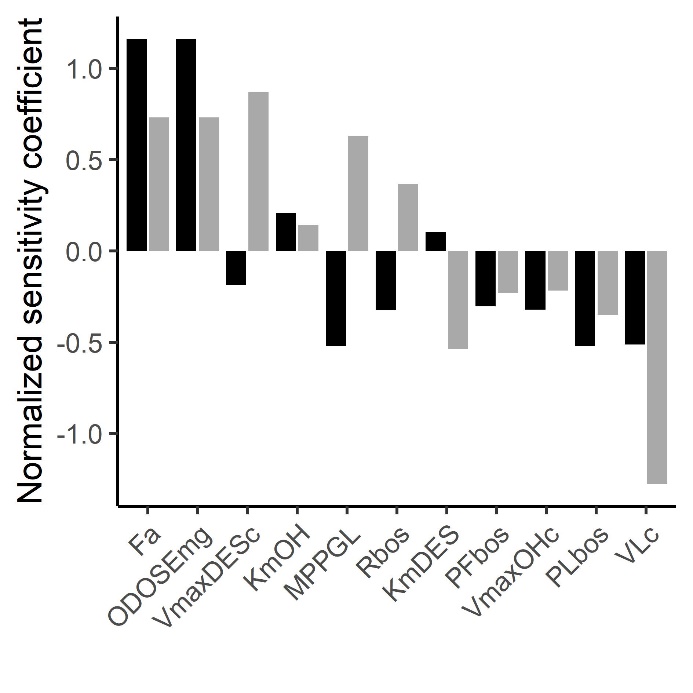


Figure S1 Sensitivity analysis of the PBK-model parameters on the predicted maximal bosentan and RO 47-8634 plasma concentrations. Black bars indicate bosentan, grey bars RO 47-8634. Only parameters with an absolute normalized sensitivity coefficient > 0.1 are shown. Fa= fraction absorbed, ODOSEmg= oral administered dose, VmaxDESc= maximal rate of RO 47-8634 formation, KmOH=Michaelis Menten constant of RO 48-5033 formation, MPPGL= microsomal protein per gram of liver, Rbos=blood:plasma ratio bosentan, KmDES=Michaelis Menten constant of RO 47-8634 formation, PFbos=fat/blood partition coefficent of bosentan, VmaxOHc=maximal rate of 48-5033 formation, PLbos= liver/blood partition coefficient of bosentan, VLc=fraction of liver tissue.

# Supplementary file II PBK model code

; Date: June 2022

; Purpose: General PBK Model GCDCA, built with in vitro and in silico derived parameter values

; Species: Human

; Compiled by: Véronique de Bruijn

; Organisation: Wageningen University

;================================================================

;Physiological parameters

;================================================================

; tissue volumes

BW = 70 {Kg} ; body weight human (variable, dependent on study)

VFc = 0.214 ; fraction of fat tissue reference: Brown et al. (1997)

VLc = 0.026 ; fraction of liver tissue reference: Brown et al. (1997)

VRc = 0.054 ; fraction of richly perfused tissue reference: Brown et al. (1997)

VSc= 0.6033 ; fraction of slowly perfused tissue reference: Brown et al. (1997)

VBc = 0.079 ; fraction of blood reference: Brown et al. (1997)

VIc = 0.009 ; fraction of intestinal tissue reference: Brown et al. (1997)

VGc= 0.0007 ; fraction of gallbladder tissue reference: Van Erpecum et al. (1992)

VLuc=0.014 ; fraction of intestinal lumen reference: Brown et al. (1997)

VF = VFc*BW {L or Kg} ; volume of fat tissue (calculated)

VL = VLc*BW {L or Kg} ; volume of liver tissue (calculated)

VR = VRc* BW {L or Kg} ; volume of richly perfused tissue (calculated)

VS = VSc*BW {L or Kg} ; volume of slowly perfused tissue (calculated)

VB = VBc* BW {L or Kg} ; volume of blood (calculated)

VI = VIc*BW {L or Kg} ; volume of intestinal tissue (calculated)

VG= VGc*BW {L or Kg} ; volume of gall bladder tissue (calculated)

VLu=VLuc*BW {L or Kg} ; volume of intestinal lumen (calculated)

;--------------------------------------------------------------------------------------------------------------------

;blood flow rates

QC = 15*BW^0.74 {L/hr} ; cardiac output reference: Brown et al. (1997)

QFc = 0.052 ; fraction of blood flow to fat tissue reference: Brown et al. (1997)

QLc = 0.046 ; fraction of blood flow to liver (excluding portal vein) reference: Brown et al. (1997)

QSc = 0.248 ; fraction of blood flow to slowly perfused tissue reference: Brown et al. (1997)

QRc=0.473 ; fraction of blood flow to richly perfused tissue reference: Brown et al. (1997)

QIc = 0.181 ; fraction of blood flow to intestines reference: Brown et al. (1997)

QF = QFc*QC {L/hr} ; blood flow to fat tissue (calculated)

QL = QLc*QC {L/hr} ; blood flow to liver tissue (calculated)

QS = QSc*QC {L/hr} ; blood flow to slowly perfused tissue (calculated)

QR = QRc*QC {L/hr} ; blood flow to richly perfused tissue (calculated)

QI = QIc*QC {L/hr} ; blood flow to intestines (calculated)

;================================================================

;Physicochemical parameters

;================================================================

;partition coefficients

logP =2.21 ; Roda 1990

RGCDCA=0.55; blood:plasma ratio, 1-Hct, assumption

PF =0.05/RGCDCA ; fat/blood partition coefficient calculated using QPPR of Rodgers & Rowland 2006

PL = 0.09/RGCDCA; liver/blood partition coefficient calculated using QPPR of Rodgers & Rowland 2006

PR = 0.125/RGCDCA ; richly perfused tissue/blood partition coefficient calculated using QPPR Rodgers & Rowland 2006

PS = 0.19/RGCDCA ; richly perfused tissue/blood partition coefficient calculated using QPPR Rodgers & Rowland 2006

PG=0.16/RGCDCA ; gut/blood partition coefficient calculated using QPPR of Rodgers & Rowland 2006

;================================================================

;Kinetic parameters

;================================================================

ka =1.047 {/hr} ;absorption rate constant from intestinal lumen to liver, fitted to experimental data Hepner (1977) and De Leon (1978)

;de novo synthesis in liver.

Ks=0.78*60*sens {umol/h/entire organ} ; reference: Kullak-Ublick (2004)

;Faecal excretion

Kf=Ks {umol/h} ; Faecal excretion equals the de novo synthesis

;biliary excretion from liver to bile canaliculi

VmaxBSEPc=5.848{umol/min/mg BSEP} ; reference: GCDCA from Kis (2009)

KmBSEP = 4.3 {umol/L} ; reference: Kis (2009)

MC=1 ; switch for Monte Carlo

aBSEPc=IF MC =1 THEN init(exp(NORMAL(-0.26, 0.403))) ELSE 0.839; BSEP protein abundance in pmoles/10^6 hepatocytes, reference Burt (2016)

aBSEP= IF aBSEPc > 0.23 AND aBSEPc < 2.58 THEN aBSEPc ELSE 0.00000001

MWBSEP=140000 ; BSEP is a 140 kDa protein, 140 000 g/mol

Hep=99 {10^6 hepatocytes/g liver} ; reference Barter (2007)

WL=20*BW {g} ; reference Soars (2002)

SF=aBSEP*MWBSEP*Hep*WL*60*10^-9 {mg BSEP/entire lever}; scaling factor, calculated

VmaxBSEP=VmaxBSEPc*SF{umol/h/entire liver}

;uncompetitive BSEP inhibition by bosentan and its metabolite desmethyl bosentan (RO 47-8634)

Kibos=12 {umol/L} ; reference: Fattinger (2001)

KiDES=8.5 {umol/L} ; reference: Fattinger (2001)

VmaxBSEPapp=VmaxBSEP/(1+CVLbos/Kibos+CVLDES/KiDES)

;distribution of bile flow excreted from liver in the bile canaliculae

QIb = 0.5 ; fraction of bile flow transported directly from liver to intestinal lumen via common bile duct reference: Molino (1986)

QGb = 1- QIb ; fraction of bile flow from liver stored in gall bladder, calculated

;systemic plasma concentration in fasting state

CBfs=2.4*sens {umol/L} ; reference: García-Cañaveras (2001)

;sensitivity individual

sens=1

;================================================================

;Run settings

;================================================================

Gdose =3020*sens{umol} ; dose in full gallbladder, Sips (2018)

dosingperiod =if time < 20 OR time > 32 AND time <44 OR time >56 AND time <68 THEN 1 else 0 ; stop gallbladder contractions during the night

;time

Starttime =8 ; in hr

Stoptime = 80; in hr

DTMIN=1E-6

DTMAX=1E-4

DTOUT=0.01

TOLERANCE=1E-12

Method Auto

;================================================================

;Model calculations

;================================================================

; gall bladder compartment

;AG = amount in the gallbladder, umol

;AG’ = Change in amount in the gallbladder, umol/hr

AG'=-pulse(AG,0, 4)*dosingperiod + VmaxBSEPapp*CVL/(KmBSEP+ CVL)*QGb

Init AG = Gdose

;-------------------------------------------------------------------------------------------------------

; liver compartment

;AL = Amount in liver tissue, umol

;AL' = Change in amount in liver tissue in time, umol/hr

AL' =QL*(CB-CVL)-VmaxBSEPapp*CVL/(KmBSEP+ CVL) + Ks + ka*ALu

CL = AL/VL

CVL = CL/PL

Init AL=0

;----------------------------------------------------------------------------------------------------------------

; intestine compartment

;ALu= amount in intestinal lumen, umol

ALu'=pulse(AG,0, 4)*dosingperiod+VmaxBSEPapp*CVL/(KmBSEP+ CVL)*QIb-Kf-ka*ALu

CLu=ALu/VLu

Init ALu=0

;AI’ = amount GCDCA in the intestinal tissue remaining, umol

AI' = QI*(CB-CVI)

Init AI = 0

CI=AI/VI

CVI=CI/PG

;-------------------------------------------------------------------------------------------------------------------

;fat compartment

;AF = Amount GCDCA in fat tissue, umol

AF' = QF*(CB-CVF)

Init AF = 0

CF = AF/VF

CVF = CF/PF

;--------------------------------------------------------------------------------------------------------------------

;tissue compartment richly perfused tissue

;AR = Amount GCDCA in richly perfused tissue, umol

AR' = QR*(CB-CVR)

Init AR = 0

CR = AR/VR

CVR = CR/PR

;--------------------------------------------------------------------------------------------------------------------

;tissue compartment slowly perfused tissue

;AS = Amount GCDCA in slowly perfused tissue, umol

AS' = QS*(CB-CVS)

Init AS = 0

CS = AS/VS

CVS = CS/PS

;--------------------------------------------------------------------------------------------------------------------

; blood compartment

;AB = Amount GCDCA in blood (umol)

AB' = QF*CVF + QL*CVL + QS*CVS + QR*CVR +QI*CVI - (QF+QL+QS+QR+QI)*CB

Init AB =0

CB = AB/VB

CBtot=CB/RGCDCA+CBfs ; concentration GCDCA in plasma, umol/L

;--------------------------------------------------------------------------------------------------------------------

; Mass balance calculations

Total =Gdose+Ks

Calculated = AL+ AS+ AR + AB + AG + AF + AI +Kf + ALu

ERROR=((Total-Calculated)/Total+1E-30)*100

MASSBBAL=Total-Calculated + 1

;Submodel bosentan and its metabolite desmethyl bosentan (RO 47-8634)

;=====================================================================

;Physiological parameters

;=====================================================================

VRcbos =(VRc+VIc) ; fraction of richly perfused tissue

VScbos=(VSc+VGc+VLuc) ; fraction of slowly perfused tissue

VRbos=VRcbos*BW ; volume of richly perfused tissue (calculated)

VSbos=VScbos*BW ; volume of slowly perfused tissue (calculated)

;--------------------------------------------------------------------------------------------------------------------

;blood flow rates

QRcbos = (QRc+QIc) ; fraction of blood flow to richly perfused tissue

QRbos=(QRcbos*QC) {L/hr} ; blood flow to richly perfused tissue (calculated)

;=====================================================================

;Physicochemical parameters

;=====================================================================

;partition coefficients

;bosentan

Rbos= 0.6 ; blood:plasma ratio (EMA 2004, Meyer 1996)

PFbos = 0.05/Rbos ; fat/blood partition coefficient calculated using QPPR of Rodgers & Rowland 2006

PLbos = 0.11/Rbos ; liver/blood partition coefficient calculated using QPPR of Rodgers & Rowland 2006

PRbos = 0.14/Rbos ; richly perfused tissue/blood partition coefficient calculated using QPPR Rodgers & Rowland 2006

PSbos = 0.21/Rbos ; richly perfused tissue/blood partition coefficient calculated using QPPR Rodgers & Rowland 2006

;metabolite RO 47-8634 (desmethyl bosentan)

RDES=0.55 ; blood:plasma ratio, assumption (1-Hct)

PFDES= 0.06/RDES ; fat/blood partition coefficient calculated using QPPR of Rodgers & Rowland 2006

PLDES= 0.15/RDES ; liver/blood partition coefficient calculated using QPPR of Rodgers & Rowland 2006

PRDES = 0.18 /RDES ; rapidly perfused/blood partition coefficient calculated using QPPR of Rodgers & Rowland 2006

PSDES = 0.30 /RDES ; slowly perfused/blood partition coefficient calculated using QPPR of Rodgers & Rowland 2006

;=====================================================================

;Kinetic parameters

;=====================================================================

;Absorption from GI-tract to liver

kabos =0.130 ; absorption {/hr}, fitted to in vivo data (Weber, 1999)

kbilebos= 23.660 ; biliary excretion bosentan {/hr}, fitted to in vivo data (Weber, 1999)

kbileDES=133.924 ; biliary excretion desmethyl bosentan {/hr}, fitted to in vivo data (Weber, 1999)

Fa=0.5 ; fraction absorbed, reference: Weber (1996)

;--------------------------------------------------------------------------------------------------------------------

;Metabolism liver

MPPGL = 32 {mg/g} ; microsomal protein per gram of liver, Barter (2007)

;based on metabolite formation of bosentan, scaled maximum rate of metapulism

;VmaxOH=maximum rate of hydroxyl bosentan formation

VmaxOHc = 16.4 {pmol/min/mg microsomal protein} ; reference Sato (2008)

VmaxOH=VmaxOHc*MPPGL*WL*60*10^-6 {umol/h/entire liver} ; calculated

; maximum rate of desmethyl bosentan formation

VmaxDESc= 7.53{pmol/min/mg microsomal protein} ; reference Sato (2008)

VmaxDES=VmaxDESc*MPPGL*WL*60*10^-6 {umol/h/entire liver} ; calculated

;metabolites of bosentan, affinity constants (umol/L)

KmOH= 6.4{umol/L} ; reference Sato (2008)

KmDES=4.8 {umol/L} ; reference Sato (2008)

;non-saturable clearance

CLOHc = 0.158 {uL/min/mg microsomal protein} ; reference Sato (2018)

CLDESc = 0.273 {uL/min/mg microsomal protein} ; reference Sato (2018)

CLOH=CLOHc*MPPGL*WL*60*10^-6 {L/h/entire liver} ; calculated

CLDES=CLDESc* MPPGL*WL*60*10^-6 {L/h/entire liver} ; calculated

;=====================================================================

;Run settings

;=====================================================================

;Molecular weight

MWbos = 551.6 ; Molecular weight bosentan

MWDES=543.6 ; Molecular weight desmethyl bosentan

;oral dose of bosentan

ODOSEmg =500 {mg } ; given oral dose in mg

ODOSEumol = ODOSEmg*1E-3/MWbos*1E6 {umol} ; given oral dose recalculated to umol

;=====================================================================

;Model calculations

;=====================================================================

; stomach compartment

;Ast = Amount bosentan remaining in stomach, umol

Ast'=pulse(ODOSEumol*Fa,-4,12)-kabos*Ast

Init Ast=0

;Ast'=-kabos*Ast

;Init Ast=ODOSEumol*Fa

;--------------------------------------------------------------------------------------------------------------------

;liver compartment

;ALbos = Amount bosentan in liver tissue, umol

ALbos' = kabos*Ast + QL*(CBbos - CVLbos)-AMOH'-AMDES'-Abilebos'

Init ALbos = 0

CLbos = ALbos/VL

CVLbos = CLbos/PLbos

;AMOH= amount metabolized to metabolite RO48-5033 (hydroxyl bosentan), umol

AMOH'= VmaxOH*CVLbos/(KmOH + CVLbos) + CLOH*CVLbos

Init AMOH=0

;AMDES = amount metabolized to metabolite RO 47-8634 (desmethyl bosentan), umol

AMDES' = VmaxDES*CVLbos/(KmDES + CVLbos)+CLDES*CVLbos

init AMDES = 0

;Abilebos= biliary excretion of bosentan

Abilebos' = kbilebos*ALbos

Init Abilebos=0

;--------------------------------------------------------------------------------------------------------------------

;fat compartment

;AFbos = Amount bosentan in fat tissue (umol)

AFbos' = QF*(CBbos-CVFbos)

Init AFbos = 0

CFbos = AFbos/VF

CVFbos = CFbos/PFbos

;--------------------------------------------------------------------------------------------------------------------

;tissue compartment richly perfused tissue

;ARbos = Amount bosentan in richly perfused tissue (umol)

ARbos' = QRbos*(CBbos-CVRbos)

Init ARbos = 0

CRbos = ARbos/VRbos

CVRbos = CRbos/PRbos

;--------------------------------------------------------------------------------------------------------------------

;tissue compartment slowly perfused tissue

;ASbos = Amount bosentan in slowly perfused tissue (umol)

ASbos' = QS*(CBbos-CVSbos)

Init ASbos = 0

CSbos = ASbos/VSbos

CVSbos = CSbos/PSbos

;--------------------------------------------------------------------------------------------------------------------

; blood compartment

;ABbos = Amount bosentan in blood (umol)

ABbos' = QL*CVLbos + QF*CVFbos+ QS*CVSbos + QRbos*CVRbos - (QL+QF+QS+QRbos)*CBbos

Init ABbos = 0

CBbos = ABbos/VB

AUCbos' = CBbos

Init AUCbos = 0

CBbosp_umol=CBbos/Rbos {umol/L} ; concentration bosentan in plasma, umol/L

CBbosp_ug=CBbosp_umol*MWbos {ug/L} ; concentration bosentan in plasma, ug/L

;=====================================================================

;Mass balance calculations

;=====================================================================

Totalbos' = pulse(ODOSEumol *Fa, -4, 12)

Init Totalbos = 1E-50

Calculatedbos = Ast + AFbos + ASbos + ARbos + ABbos + ALbos +AMOH + AMDES+Abilebos

ERRORbos=((Totalbos-Calculatedbos)/Totalbos+1E-30)*100

MASSBBALbos=Totalbos-Calculatedbos + 1

;=====================================================================

;desmethyl bosentan (RO 47-8634)

;=====================================================================

;ALDES = amount remaining in liver of metabolite RO 47-8634 (desmethyl bosentan) (umol)

ALDES'=QL*(CBDES-CVLDES)+AMDES'-AbileDES'

Init ALDES=0

CLDES=ALDES/VL

CVLDES=CLDES/PLDES

;AbileDES=amount desmethyl bosentan excrted via bile, umol

AbileDES'=kbileDES*ALDES

Init AbileDES=0

;--------------------------------------------------------------------------------------------------------------------

;fat compartment

;AFDES = Amount Desmethyl bosentan in fat tissue (umol)

AFDES' = QF*(CBDES-CVFDES)

Init AFDES = 0

CFDES = AFDES/VF

CVFDES = CFDES/PFDES

;--------------------------------------------------------------------------------------------------------------------

;tissue compartment richly perfused tissue

;ARDES = Amount Desmethyl bosentan in richly perfused tissue (umol)

ARDES' = QRbos*(CBDES-CVRDES)

Init ARDES = 0

CRDES = ARDES/VRbos

CVRDES = CRDES/PRDES

;--------------------------------------------------------------------------------------------------------------------

;tissue compartment slowly perfused tissue

;ASDES = Amount Desmethyl bosentan in slowly perfused tissue (umol)

ASDES' = QS*(CBDES-CVSDES)

Init ASDES = 0

CSDES = ASDES/VSbos

CVSDES = CSDES/PSDES

;--------------------------------------------------------------------------------------------------------------------

; blood compartment

;ABDES = Amount Desmethyl bosentan in blood (umol)

ABDES' = QL*CVLDES + QF*CVFDES+ QS*CVSDES + QRbos*CVRDES - (QL+QF+QS+QRbos)*CBDES

Init ABDES = 0

CBDES = ABDES/VB

AUCDES' = CBDES

Init AUCDES = 0

CBDESp_umol=CBDES/RDES {umol/L} ; concentration desmethyl bosentan in plasma, umol/L

CBDESp_ug=CBDESp_umol*MWDES {ug/L} ; concentration desmethyl bosentan in plasma, ug/L

;=====================================================================

;Mass balance calculations

;=====================================================================

TotalDES'=AMDES'

Init TotalDES=1e-50

CalculatedDES=AFDES+ARDES+ABDES+ALDES+ASDES+AbileDES

ERRORDES=((TotalDES-CalculatedDES)/TotalDES+1E-30)*100

MASSBBALDES=TotalDES-CalculatedDES + 1

**References**

Barter, Z. E., Bayliss, M. K., Beaune, P. H., Boobis, A. R., Carlile, D. J., Edwards, R. J., ... & Rostami-Hodjegan, A. (2007). Scaling factors for the extrapolation of in vivo metabolic drug clearance from in vitro data: reaching a consensus on values of human micro-somal protein and hepatocellularity per gram of liver. *Current drug metabolism*, *8*(1), 33-45.

Brown RP, Delp MD, Lindstedt SL, Rhomberg LR, Beliles RP (1997) Physiological parameter values for physiologically based pharmacokinetic models. Toxicol Ind Health 13(4):407-84

Burt, H. J., Riedmaier, A. E., Harwood, M. D., Crewe, H. K., Gill, K. L., & Neuhoff, S. (2016). Abundance of hepatic transporters in Caucasians: a meta-analysis. Drug Metabolism and Disposition, 44(10), 1550-1561.

De Leon, M. P., Murphy, G. M., & Dowling, R. H. (1978). Physiological factors influencing serum bile acid levels. *Gut*, *19*(1), 32-39.

EMA (2004). Scientific discussion. Retrieved from: https://www.ema.europa.eu/en/documents/scientific-discussion/tracleer-epar-scientific-discussion_en.pdf

Fattinger, K., Funk, C., Pantze, M., Weber, C., Reichen, J., Stieger, B., & Meier, P. J. (2001). The endothelin antagonist bosentan inhibits the canalicular bile salt export pump: a potential mechanism for hepatic adverse reactions. Clinical Pharmacology & Therapeutics, 69(4), 223-231.

García-Cañaveras, J. C., Donato, M. T., Castell, J. V., & Lahoz, A. (2012). Targeted profiling of circulating and hepatic bile acids in human, mouse, and rat using a UPLC-MRM-MS-validated method. *Journal of lipid research*, *53*(10), 2231-2241.

Hepner, G. W., & Demers, L. M. (1977). Dynamics of the enterohepatic circulation of the glycine conjugates of cholic, chenodeoxycholic, deoxycholic, and sulfolithocholic acid in man. *Gastroenterology*, *72*(3), 499-501.

Hofmann, A. F. (1999). Bile acids: the good, the bad, and the ugly. *Physiology*, *14*(1), 24-29.

Hofmann, A. F., Molino, G., Milanese, M., & Belforte, G. (1983). Description and simulation of a physiological pharmacokinetic model for the metabolism and enterohepatic circulation of bile acids in man. Cholic acid in healthy man. *The Journal of clinical investigation*, 71(4), 1003-1022.

Kis, E., Ioja, E., Nagy, T., Szente, L., Heredi-Szabo, K., & Krajcsi, P. (2009). Effect of membrane cholesterol on BSEP/Bsep activity: species specificity studies for substrates and inhibitors. *Drug metabolism and disposition*, 37(9), 1878-1886.

Kullak-Ublick, G. A., Stieger, B., & Meier, P. J. (2004). Enterohepatic bile salt transporters in normal physiology and liver disease. *Gastroenterology*, *126*(1), 322-342.

Meyer, R., J. . (1996). In vitro binding of the endothelin receptor antagonist ro 47-0203 to plasma proteins in man and animals, and red blood cell/plasma partitioning. *Basel F: Hoffmann-La Roche Ltd*.

Roda, A., Minutello, A., Angellotti, M. A., & Fini, A. (1990). Bile acid structure-activity relationship: evaluation of bile acid lipophilicity using 1-octanol/water partition coefficient and reverse phase HPLC. *Journal of lipid research*, *31*(8), 1433-1443.

Rodgers, T., & Rowland, M. (2006). Physiologically based pharmacokinetic modelling 2: predicting the tissue distribution of acids, very weak bases, neutrals and zwitterions. Journal of pharmaceutical sciences, 95(6), 1238-1257.

Sato, M., Toshimoto, K., Tomaru, A., Yoshikado, T., Tanaka, Y., Hisaka, A., ... & Sugiyama, Y. (2018). Physiologically based pharmacokinetic modeling of bosentan identifies the saturable hepatic uptake as a major contributor to its nonlinear pharmacokinetics. Drug Metabolism and Disposition, 46(5), 740-748.

Sips, F. L., Eggink, H. M., Hilbers, P. A., Soeters, M. R., Groen, A. K., & Van Riel, N. A. (2018). In silico analysis identifies intestinal transit as a key determinant of systemic bile acid metabolism. Frontiers in physiology, 9, 631.

Soars, M. G., Burchell, B., & Riley, R. J. (2002). In vitro analysis of human drug glucuronidation and prediction of in vivo metabolic clearance. *Journal of Pharmacology and Experimental Therapeutics*, *301*(1), 382-390.

Van Erpecum, K. J., Henegouwen, G. P. V. B., Stolk, M. F., Hopman, W. P., Jansen, J. B., & Lamers, C. B. (1992). Fasting gallbladder volume, postprandial emptying and cholecystokinin release in gallstone patients and normal subjects. *Journal of hepatology*, *14*(2-3), 194-202.
